# Idiopathic fibrotic lung disease at a university hospital setting: management and prognostic factors

**DOI:** 10.3402/ecrj.v2.26915

**Published:** 2015-03-24

**Authors:** Parya Rad, Carl-Axel Karlsson, Christer Janson

**Affiliations:** Department of Medical Sciences, Respiratory, Allergy and Sleep Research, Uppsala University, Uppsala, Sweden

**Keywords:** fibrotic lung disease, idiopathic pulmonary fibrosis, diagnostics, prognosis, survival

## Abstract

**Background:**

Idiopathic fibrosing interstitial pneumonia consists of many subtypes, most associated with a poor prognosis. The aim of the study was to evaluate diagnostic procedures and treatment as well as survival in patients with idiopathic fibrosing interstitial pneumonia.

**Methods:**

This study comprised 175 patients with idiopathic fibrosing interstitial pneumonia (ICD 10 code J84) that had been diagnosed at Uppsala University Hospital, during 2005 to 2012. Patient records were reviewed concerning: gender, age, smoking, occupational exposure, comorbidities, procedures, lung function, and treatment. Information on survival and cause of death was collected.

**Results:**

A total of 98% had been examined with computed tomography, 93% with spirometry, 49% with measurement of diffusion capacity, 48% with bronchoalveolar lavage, and 23% with lung biopsy. Prednisolone had been prescribed to 74% while *N*-acetylcysteine (NAC) and omeprazole were prescribed to 54%, respectively. Five-year survival was 46%. Mortality was associated with high age, low diffusion capacity, and the use of NAC.

**Conclusion:**

High age and a low diffusion capacity are related to shorter survival in idiopathic fibrosing interstitial pneumonia. We also unexpectedly found that the use of NAC was related to shorter survival. A relatively low proportion of the patients were examined with diffusion capacity measurement. Thus, there is a possibility to improve diagnostic procedures and thereby improve estimation of prognosis in fibrotic lung disease.

Idiopathic fibrosing interstitial pneumonia consists of several subgroups, the most common form being idiopathic pulmonary fibrosis (IPF) (1). Other subgroups in this disease group include idiopathic non-specific interstitial pneumonia, desquamative interstitial pneumonia, cryptogenic organizing pneumonia, and acute interstitial pneumonia (2). The main common feature is the development of a restrictive lung function and impaired carbon dioxide diffusion capacity (DL_CO_). Furthermore, for most patients the primary symptom is progressive effort dyspnoea as well as non-productive cough. The tissue damage is mainly localized in the lung's interstitium, and the space between the capillary endothelium and alveolar epithelium (3).

It is recommended that a multidisciplinary approach including pulmonary clinicians, pathologists and radilogists in joint rounds is used in the diagnostic procudure of fibrotic lung disease (1, 3, 4). Previously major and minor criteria were used in the diagnosis of IPF. If the criteria for a patient were not met lung biopsy was recommended (5). During the last few years, the expertise of the subject has concluded that a classical UIP-pattern on high-resolution computed tomography (HRCT) in combination with typical clinical findings is sufficient for the diagnosis IPF (1). UIP is characterized on HRCT by the presence of reticular opacities, often associated with traction bronchiectasis. Honeycombing is common and is critical for making a definite diagnosis. In some patients surgical lung biopsies are needed to make the diagnosis (1).

Treatment guidelines for fibrosing interstitial pneumonia have mainly been developed for IPF. Pulmonary transplantation is, in the current situation, the only treatment that can provide a significant long-term survival in IPF (1). Treatment with pirfenidone has been shown to lead to a slower decline in lung function and 6 min walking test and render a longer progression-free survival (6, 7). Recently, there has also been data indicating a positive effect of pirfenidone on overall mortality (7). Previous treatment guidelines of IPF included the use of prednisolone together with immunosuppressive agents such as cyclophosphamide and azathioprine (5). These recommendations have, however, recently been revised as this kind of treatment has been shown to have a negative effect on survival in IPF (8). It has been suggested that *N*-acetylcysteine (NAC) given at high doses has a beneficial effect on lung function in IPF (9); however, a recent randomized controlled trial failed to show such an effect (10). A high prevalence of gastroesophageal reflux has been found among patients with IPF (11) and some studies indicate that antacid treatment may have a positive effect in IPF (12, 13).

In fibrosing interstitial pneumonia, the radiologic pattern non-specific interstitial pneumonia (NSIP) is related to better survival compared to usual interstitial pneumonia (UIP) (3, 14). Shorter survival in IPF has been related to high age, male gender, low forced expiratory volume FVC, a high ratio of forced expiratory volume in one second (FEV_1_) and FVC (FEV_1_/FVC), and low diffusion capacity (DL_CO_) (15–17). A higher number of macrophages in bronchoalveolar fluid (BAL) has also been associated with shorter survival (14).

The objectives of the study were to evaluate diagnostic procedures and treatment as well as finding prognostic factors of survival in patients with idiopathic fibrosing interstitial pneumonia.

## Method

The study population comprised all patients that had been diagnosed with idiopathic fibrosing interstitial pneumonia (IC10 code J84) at the Department of Respiratory and Allergy Disease at the University Hospital Uppsala during the period 2005 to 2012. In total 177 patients were included. Two patients that got their diagnosis on the day that they deceased were excluded.

Information on the following variables was collected through the patient's records: age at time of diagnosis, ICD 10 codes, smoking status, occupation, radiological examination, and other diagnostic procedures.

Results regarding the following lung function variables were collected: FVC, FEV_1_, FEV_1_/FVC, and DL_CO_.

Results from analyses of blood samples were recorded for rheumatoid factor, anti-nuclear antibodies (ANA), anti-neutrophil cytoplasmic antibodies with cytoplasmic pattern (C-ANCA), and anti-neutrophil cytoplasmic antibodies with perinuclear pattern (P-ANCA). Cell counts from bronchoalveolar lavage (BAL) were recorded.

The following treatment options were recorded: NAC, prednisolone, azathioprine, cyclophosphamide, omeprazole, pirfenidone, and lung transplantation.

Information on mortality and cause of death was obtained from the Swedish Cause of Death Register for the period 1 January 2005 to 1 September 2013.

An ethical approval of the study was obtained from The Regional Ethical Review Board in Uppsala, diary number 2013/337.

### Statistics

Descriptive statistic methods were used to describe diagnostic procedures, examination results, and treatments. Cox regression was used to study the impact of different factors upon survival. Hazard risk ratios with a confidence interval of 95% were calculated. Correlations with *p*<0.05 were considered to be significant.

## Results

The majority of the 175 included patients were men and J84.1, which includes IPF, was the dominating diagnosis code. Many of the patients were previously smokers and comorbidities were common (Table 1).

**Table 1:** Age, sex, diagnoses, smoking status, exposition (mean±SD and *n* (%))

Age	68±13
Women	64 (37)
Diagnoses	
J84.0 Alveolar and parietoalveolar conditions	5 (3)
J84.1 Other interstitial pulmonary diseases with fibrosis	136 (78)
J84.8 Other specified interstitial pulmonary diseases	20 (11)
J84.9 Interstitial pulmonary disease, unspecified	57 (33)
Smoking	
Never	65 (38)
Previous	98 (57)
Current	10 (6)
Occupations with exposition for airway irritants^a^	69 (41)
Co-morbidities	
Essential hypertension	85 (49)
Ischemic heart disease	48 (27)
Rheumatic diseases	43 (25)
Diabetes mellitus	40 (23)
COPD	29 (17)
Cerebrovascular diseases	13 (7)

a Available in 170 subjects.

**Fig. 1:**
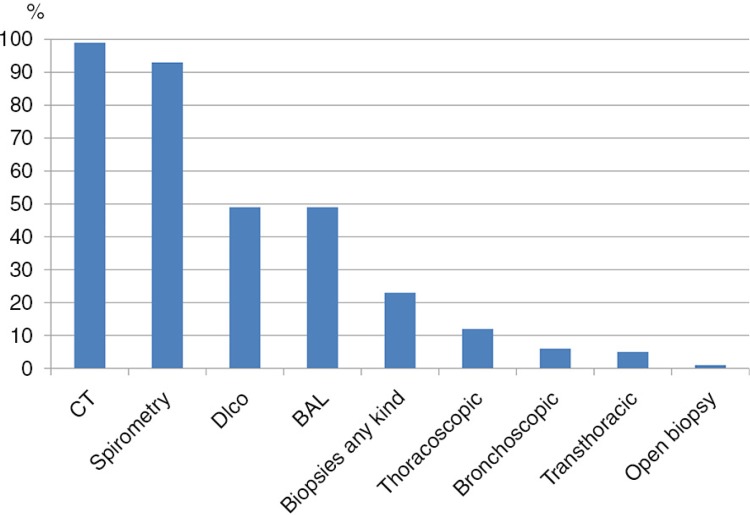
Diagnostic procedures in the patient cohort.

Computed tomography and spirometry had been performed in almost all patients, DL_CO_ and BAL in almost half and biopsies in about one fourth of the patients (Fig. 1). During the study period, the proportion of patients undergoing a biopsy had decreased from 37% in those diagnosed before 2009 to 14% in those diagnosed in 2011 and 2012 (*p*=0.01). Assessment of DL_CO_ also declined from 57% before 2009 to 34% in 2011 and 2012 (*p*=0.02). No significant time trend was seen for any other diagnostic procedure.

Mean FVC was about 70% of predicted value and DL_CO_ was 50% of the predicted value at diagnosis. Macrophages were the dominating cell type in BAL (Table 2). A positive rheumatoid factor was found in 40% of the 76 patients where this was measured, a positive antinuclear antibody was found in 27%, while perinuclear anti-neutrophil cytoplasmic antibodies (P-ANCA) in 7%, and C-ANCA in 5% was found in patients where such a measurement was performed (*n*=75).

**Table 2:** Pulmonary function (% of predicted) and cell count (%) in bronchoalveolar lavage

	Mean±SD	Range
Lung function		
FVC	71±18	28–125
FEV_1_/VC	107±15	50–139
DL_CO_	52±17	20–100
BAL		
Macrophages	62±25	6–98
Neutrophilic granulocytes	12±16	0–84
Eosinophilic granulocytes	4±9	0–68
Lymphocytes	22±21	0–94
Monocytes	1±3	0–14

**Fig. 2:**
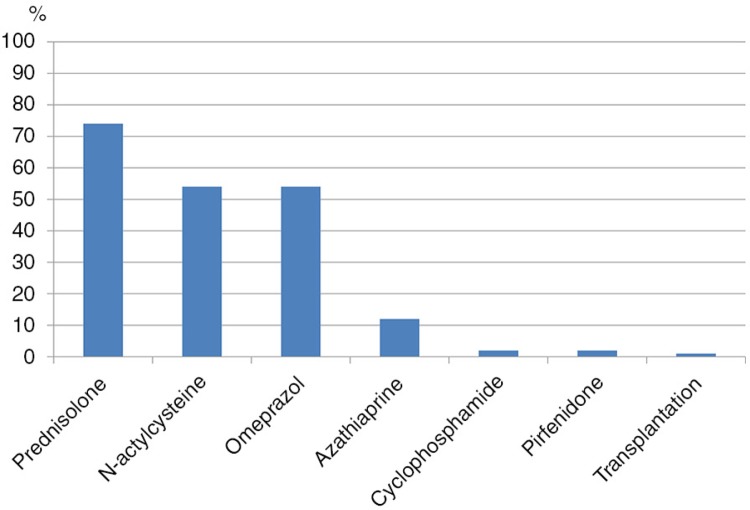
Treatments used in the patient cohort.

Prednisolone, NAC, and omeprazole were the dominating pharmacological treatments (Fig. 2). Of the patients that had used NAC, 24 (25%) had used high-dose treatment, 600 mg three times daily.

**Fig. 3:**
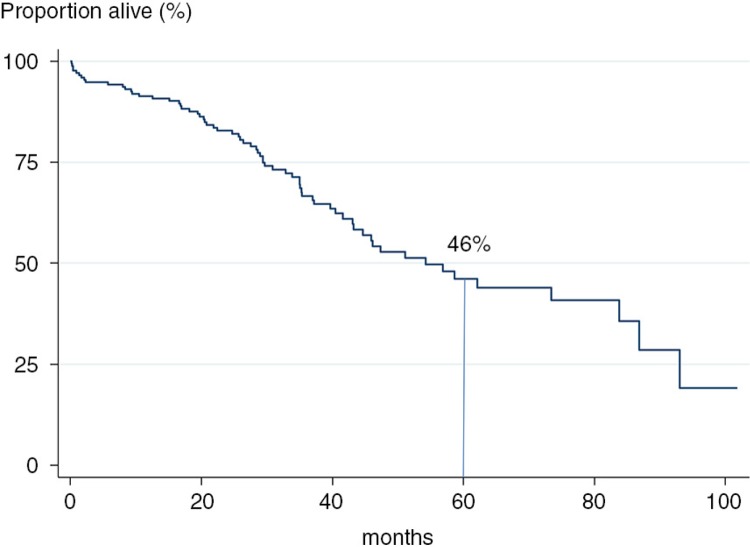
Survival in the patient cohort.

Of the 175 patients followed, 67 (38%) died during the time of the study. This corresponded to a 5-year survival of 46% (Fig. 3). The cause of death was available for 59 patients. In a majority, the primary cause of death was airway-related disease (63%) while 15% died of cancer and 14% of cardiovascular disease.

The factors shown to be statistically significant to mortality in the univariate analyses were age, smoking status, ischemic heart disease, DL_CO_, and treatment with NAC (Table 3). After adjustment for age, DL_CO_ and use of high doses of NAC remained significantly associated with mortality (Table 3).

**Table 3:** Variables associated with mortality in the patient cohort

	Unadjusted	Adjusted^a^
	HRR (95% CI)	HRR (95% CI)
Age (per 10 years)	1.92 (1.49–2.48)	1.91 (1.45–2.52)
Smoking (ever)	1.97 (1.14–3.40)	1.24 (0.71–2.18)
Ischemic heart disease	2.16 (1.30–3.59)	1.09 (0.64–1.86)
DL_CO_ (10 units)	0.70 (0.55–0.89)	0.72 (0.35–0.97)
*N*-Acetylcysteine		
Low dose	2.15 (1.22–3.89)	1.74 (0.98–3.09)
High dose	2.60 (1.27–5.30)	2.88 (1.37–6.08)

HRR, hazard risks ratio.

a adjusted for age and smoking status.

## Discussion

The main finding of this study is that idiopathic fibrosing interstitial pneumonia is a condition with a high mortality with less than 50% of the patients alive after a 5-year period. High age and low diffusion capacity were independently related to mortality. Surprisingly enough, we also found that high-dose treatment with NAC was associated with an almost three times higher risk of dying in idiopathic fibrotic lung disease.

This study was a retrospective study based on a review of patient records. It therefore has several methodological weaknesses especially as it covers a time period where the diagnostic procedures and management of fibrotic lung diseases have undergone substantial changes. In this study it is also not possible to distinguish IPF from the other kinds of idiopathic fibrotic lung diseases. Nevertheless, we believe that this kind of real-life study may shed some light on how these kinds of patients are managed in everyday clinical practice.

As expected nearly all patients had undergone CT and spirometry during the diagnostic phase, but only half had a measurement of diffusion capacity. An increased use of DL_CO_ should be encouraged as this is a non-invasive method which has been shown to be of considerable prognostic value in IPF (14, 16). On the other hand, almost half of the patients had undergone BAL, which seems like a rather high figure since BAL is not recommended as a routine method in the majority of patients with idiopathic fibrosing interstitial pneumonia (1). A likely explanation to the high use of BAL is that this was possibly recommended as a procedure for excluding other diagnoses in previous IPF guideline that were not replaced until 2011 (5). Multidisciplinary rounds are advocated in the management of fibrotic lung disease (18). Such rounds have been used at the department since 2011, but unfortunately we were unable to find this information in our review of the patient's records.

Prednisolone, NAC and omeprazole were the most commonly used pharmacological treatments. The pharmacological management of IPF, in particular, is undergoing large changes with some medications such as prednisolone and NAC shown to be of questionable value (8, 10) while the evidence is increasing for the benefit of other treatment options such as pirfenidone (7). There are also new drugs being tested that may further improve the prognosis of IPF (19).

Five-year survival was 46% in the present study; this is comparable to what Gribbin et al. reported where the 3- and 5-year survival in IPF was 57 and 43%, respectively (20). High age and low DL_CO_ were associated with shorter survival, as expected (14, 15). Comorbidities were common among the patients and in the unadjusted analysis having ischemic heart disease was associated with higher mortality. This association disappeared after adjusting for age which may imply that part of the association between age and mortality in IPF is mediated through comorbidities such as ischemic heart disease.

One theory of why treatment with NAC correlated with a poor prognosis in this study could be that patients with repeated exacerbations are more often prescribed the drug, as it is regarded as a relatively harmless and simple treatment. However, prednisolone is also often prescribed primarily during periods of exacerbations and no association was found between the use of prednisolone and mortality. It was also notable that the highest risk of mortality was found in patients receiving high-dose treatment. Another possibility is that high-dose NAC treatment primarily has been used in IPF patients, and IPF has a poorer prognosis than the other forms of idiopathic fibrotic lung disease. An analysis of the relationship between NAC treatment and mortality in those with IPF would therefore be warranted. Unfortunately, it was not possible to distinguish IPF from the other kinds of idiopathic fibrotic lung diseases in the present study.

The conclusion of this study is that higher age and low diffusion capacity for carbon monoxide is associated with shorter survival in interstitial lung diseases. We also found an unexpected negative correlation between use of NAC and survival. Relatively few patients had been examined with DL_CO_. Thus, there is a possibility to improvediagnostic procedures and thereby improve estimation of prognosis in idiopathic fibrosing interstitial pneumonia.
